# Transneuronal Degeneration of Thalamic Nuclei following Middle Cerebral Artery Occlusion in Rats

**DOI:** 10.1155/2016/3819052

**Published:** 2016-08-11

**Authors:** Shu-Jen Chang, Juin-Hong Cherng, Ding-Han Wang, Shu-Ping Yu, Nien-Hsien Liou, Ming-Lun Hsu

**Affiliations:** ^1^School of Dentistry, National Yang-Ming University, No. 155, Section 2, Li-Non Street, Taipei 112, Taiwan; ^2^Department and Graduate Institute of Biology and Anatomy, National Defense Medical Center, No. 161, Section 6, Minquan E. Road, Taipei 114, Taiwan

## Abstract

*Objective.* Postinfarction transneuronal degeneration refers to secondary neuronal death that occurs within a few days to weeks following the disruption of input or output to synapsed neurons sustaining ischemic insults. The thalamus receives its blood supply from the posterior circulation; however, infarctions of the middle cerebral arterial may cause secondary transneuronal degeneration in the thalamus. In this study, we presented the areas of ischemia and associated transneuronal degeneration following MCAo in a rat model.* Materials and Methods.* Eighteen 12-week-old male Sprague-Dawley rats were randomly assigned to receive middle cerebral artery occlusion surgery for 1, 7, and 14 days. Cerebral atrophy was assessed by 2,3,5-triphenyltetrazolium hydrochloride staining. Postural reflex and open field tests were performed prior to animal sacrifice to assess the effects of occlusion on behavior.* Results.* Myelin loss was observed at the lesion site following ischemia. Gliosis was also observed in thalamic regions 14 days following occlusion. Differential degrees of increased vascular endothelial growth factor expression were observed at each stage of infarction. Increases in myelin basic protein levels were also observed in the 14-day group.* Conclusion.* The present rat model of ischemia provides evidence of transneuronal degeneration within the first 14 days of occlusion. The observed changes in protein expression may be associated with self-repair mechanisms in the damaged brain.

## 1. Introduction

The brain and spinal cord consist of neurons with different functions, such as movement control and sensory information processing. The loss of function of neurons in the brain and spinal cord is known as neurodegeneration [1, 2]. Current research suggests that transneuronal degeneration plays a significant role in a number of neurodegenerative diseases, including Alzheimer's disease, Parkinson's disease, Huntington's disease, and amyotrophic lateral sclerosis [3–7].

Transneuronal degeneration is also referred to as secondary neuronal degeneration and includes the following four stages: axonal transection, anterograde degeneration, retrograde degeneration, and associated neuronal degeneration [7]. In the human brain, the pathological processes induced by physical trauma or vascular blockages may cause neuronal damage, resulting in a disruption of axonal transmission in which both input and output information cannot be received by the associated neurons, following which anterograde and retrograde degeneration occur.

Ischemic stroke, also known as cerebral infarction, may occur at the bifurcation of the middle cerebral artery (MCA). The MCA, supplying 80% of the blood supply to the brain, is the largest branch of the internal carotid artery (ICA) and the most common site for the occurrence of cerebral embolism. Research has revealed that severe ischemic stroke in the MCA results in damage and eventual necrosis of neurons in the striatum and substantia nigra due to the resulting disruption in blood supply. Saji and Reis, for example, reported that MCA occlusion (MCAo) leads to decreased GABAergic input to the substantia nigra (SN) as well as elevated blood flow, metabolism, and neuronal activity in SN neurons [8]. Loss of inhibitory GABAergic innervation in the SN following striatal lesions shifts the balance between inhibitory and excitatory input, leading to a state of excessive excitation that ultimately results in neuronal cell death.

Though a number of studies have confirmed that MCAo results in damage to neurons directly supplied by the MCA, researchers have also documented the occurrence of secondary neuronal degeneration in areas with synaptic connections to the ischemic regions, including the thalamus and substantia nigra [9, 10]. Hata et al. further reported that, following MCAo, brain damage may also occur in deep regions connected to the neuronal structures supplied by the MCA, such as the hippocampus, hypothalamus, and globus pallidus, as well as in the substantia nigra ipsilateral to striatal infarction [11]. This form of neuronal atrophy, known as anterograde transneuronal degeneration, occurs following the deafferentation of postsynaptic neurons. However, the mechanisms underlying transneuronal degeneration in remote areas remain poorly understood. In the present study, we investigated areas of ischemia and associated transneuronal degeneration following MCAo in a rat model of cerebral ischemia.

## 2. Materials and Methods

### 2.1. Surgical Occlusion of the Middle Cerebral Artery

All experimental procedures of the present study were approved by the Institutional Animal Care and Use Committee of the National Defense Medical Center. All efforts were made to minimize the number of animals utilized and their suffering with Guide for Care and Use of Laboratory Animals. Each group consisted of six male Sprague-Dawley rats (250–300 g) exposed to MCAo for 1 d, 7 d, and 14 d and sham control.

**Figure 1:**
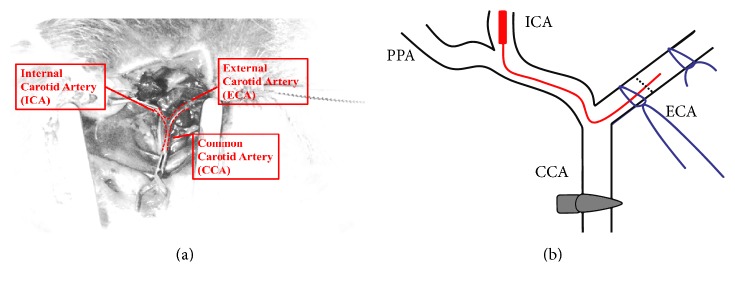
Illustration of middle cerebral artery occlusion procedure. (a) The sternocleidomastoid muscle and sternohyoid gap are located in the vicinity of the right common carotid artery, dividing in the neck to form the external and internal carotid arteries. (b) A 4-0 nylon suture (red line) is inserted and moved towards the internal carotid artery bifurcation, and the blood supply from the external carotid artery is cut off. The 4-0 nylon suture is inserted 20–22 mm into the internal carotid artery in order to slowly occlude the middle cerebral artery. CCA: common carotid artery, ECA: external carotid artery, ICA: internal carotid artery, and PPA: pterygopalatine artery.

In the present study, we simulated human stroke by inducing focal cerebral ischemia in rats. MCAo was performed according to the experimental methods published by Uluç et al. in 2011 [12]. Prior to the operation, a 22 mm, 4-0 size nylon suture (UNIK Surgical Sutures MFG, Co., Taiwan) was prepared, and its tip was wrapped with Parafilm (Parafilm M®, Bemis, Germany) approximately 1 mm long and 0.1 cm thick. Following Zoletil (20 mg/kg) anesthetization, an approximately 4-cm incision was made in the center of the neck, and the right sternoclavicular mastoid muscle was exposed. The right external carotid artery (ECA) and ICA were located by following the right common carotid artery to the brain (Figure 1(a)). The common carotid artery was fixed with an artery clamp, and an incision was made in the ECA. The prepared 4-0 nylon suture was pushed forward from the incision to the bifurcation of the ECA and ICA. The ECA was ligated and incised (Figure 1(b)). The 4-0 nylon suture was pushed forward between 20 mm and 22 mm into the ICA until the tip reached the MCA in order to complete the occlusion procedure. The ECA and skin incisions were then sutured closed, and the animals were allowed to wake from the anesthesia under a heat lamp. All animals were provided free access to food and water.

### 2.2. Postural Hang Reflex and Open Field Test

Developed by Bederson et al. [13, 14], the postural hang reflex test was performed in order to evaluate sensorimotor function via assessment of upper body posture. The degree of abnormal posture was estimated by suspending the rats by their tails 100 cm above the floor and slowly lowering them. Mild cases of abnormal posture were defined by flexing the contralateral limb towards the body, while more severe cases were defined by circling towards the ipsilateral side. In addition, each animal individually underwent a single, 3-minute open field test for the assessment of local motor activity.

Both tests were scored according to the following system. A score of zero indicated normal motor activity; a score of 1 was given when the body is consistently bent towards the side contralateral to the occlusion; a score of 2 was given when animal was suspended by its tail, leading to circling behavior of the side contralateral to the occlusion; a score of 3 was given when rats exhibited winding behavior towards the side contralateral to the occlusion; a score of 4 was given when rats exhibited difficulty in/loss of the ability to walk (Table 1).

### 2.3. Brain Sections Preparation

Brains were removed at various times after reperfusion (MCAo: 1 d, 7 d, and 14 d). The brains of MCAo rats were dissected and sliced using a matrix device (Plastics One Inc., USA) into 2 mm coronary sections. After mediosagittal division, the main portion of the infarct was snap frozen and stored for further analyses.

### 2.4. TTC Staining

After sectioning, slices were immediately stained with 1% TTC (triphenyltetrazolium hydrochloride, USB Corporation, USA) in 0.9% NaCl at 37°C for 15 min. The sections were then transferred to 10% formaldehyde (Hayashi Pure Chemical IND, LTD, Japan) for 16 h in order to complete fixation.

### 2.5. Measurement of Infarct Volume

Five equidistant coronal sections 2 mm from bregma +4.0 mm to bregma −7.0 mm were stained with TTC and photographed using a Leica vertical microscope (DM2500, Wetzlar, Germany). The area of the infarct was determined in each slice (Image J 1.45, Wayne Rasband, USA). The infarct volume was calculated by multiplying the sum of the area by the distance between each section. To account for differential shrinkage resulting from tissue processing, the infarct sizes were measured as follows: corrected injury volume = (measured bilateral hemisphere volume − injury volume)/bilateral hemisphere volume.

### 2.6. Luxol Fast Blue-Cresyl Echt Violet Stain

Brain sections were dehydrated with 75% alcohol and subsequently incubated in Luxol fast blue stain solution at 25°C for 24 h. Sections were then washed with distilled water and quickly dipped in 0.05% lithium carbonate and 70% regent alcohol for gray and white matter differentiation. The sections were then incubated in 0.1% cresyl echt violet stain for 3 min, dipped in 70% regent alcohol 5–10 times, dehydrated through three changes of absolute alcohol, and finally mounted with Permount medium (Thermo Fisher Scientific, USA). The white matter bundles were examined using light microscopy (Zeiss Axio Imager A2, Germany).

### 2.7. Immunohistochemistry

Brain samples were harvested and kept in 4% paraformaldehyde for 24 h and immersed in 30% sucrose for 3-4 days at 4°C. After cryoprotection in 30% sucrose, the brains were rapidly frozen in isopentane and stored at −80°C. Twenty micrometer cryostat sections at the level of the thalamus (bregma −3.3 mm) according to a stereotaxic atlas [15] were processed for immunohistochemistry. Immunohistochemistry analyses were performed with the UltraVision Quanto Detection System HRP DAB kit (Thermo Fisher Scientific, USA). The primary antibodies utilized were rabbit anti-GFAP antibody (glial fibrillary acidic protein, Santa Cruz Biotechnology, USA, 1 : 200 dilution), anti-VEGF (vascular endothelial growth factor, Millipore Corporation, USA, 1 : 400), and anti-MBP (myelin basic protein, Santa Cruz Biotechnology, USA, 1 : 200). The secondary antibodies utilized were biotinylated goat anti-mouse IgG and goat anti-rabbit IgG (Thermo Fisher Scientific, USA, 1 : 400 dilution). The results were observed using the Leica vertical microscope.

## 3. Results

### 3.1. Locomotor Behavior following MCA Occlusion

**Figure 2:**
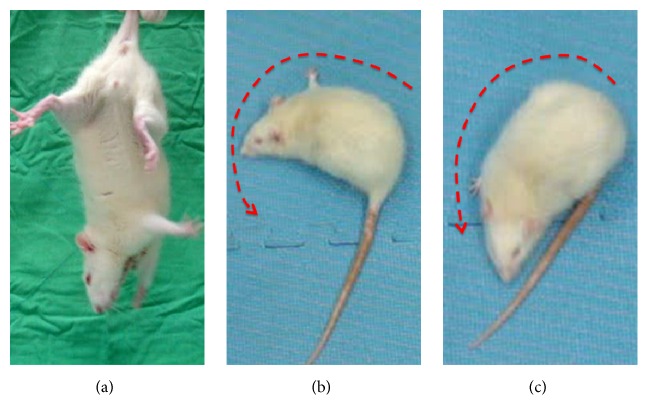
Confirmation of locomotor activity in rats on postsurgery day 1. (a) The body of the rat bent consistently to the side contralateral to that of the damaged brain hemisphere. (b, c) The rat could only circle to the side contralateral to that of the damaged brain hemisphere.

The locomotor behavior of the animals was tested on the 1st, 7th, or 14th day after surgery, depending on the group to which the rats had been assigned. On the first day after surgery, rats consistently bent their bodies to the side contralateral to that of the damaged brain hemisphere during the postural hang reflex test (Figure 2(a)). In the open field test, the 1-day rats were still able to move, though this movement was restricted to a circular pattern towards the side contralateral to that of the damaged brain hemisphere (Figures 2(b) and 2(c)). Behavioral tests were conducted again on the 7th and 14th days following surgery for the remaining two groups. Though rats in the 7-day group no longer exhibited the contralateral circling behavior, their bodies still bent to the side contralateral to that of the damaged brain hemisphere in the tail suspension test at a rate of 70%. Rats in the 14-day group exhibited no significant differences in behavior from normal rats; though the alignment of their body axes had changed. Contralateral bending again occurred at a rate of 70%.

### 3.2. Analysis of Brain Mitochondrial Dehydrogenase Activity and Cerebral Infarct Area

**Figure 3:**
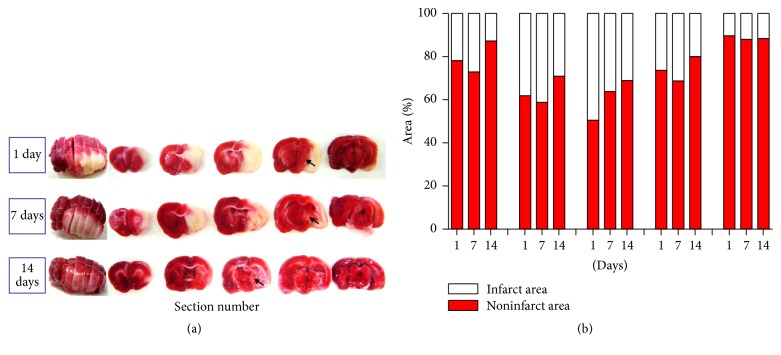
Results of 2,3,5-triphenyltetrazolium chloride staining and calculation of the infract area. (a) Results of 2,3,5-triphenyltetrazolium chloride staining in the 1-day, 7-day, and 14-day groups. The arrow indicates the thalamus. (b) Quantification of the staining results and percentage of cerebral infarct area. Infarction was most severe at 7 days after surgery.

TTC (2,3,5-triphenyltetrazolium chloride solution) is a colorless solution that undergoes a reduction reaction with dehydrogenase in the mitochondria of normal cells. After MCAo, no reaction was observed between TTC and mitochondrial dehydrogenase in the damaged brain tissue; therefore, the cerebral ischemic area appeared white after TTC staining (Figure 3(a)). In the 7-day group, the left and right hemispheres differed in size, with the damaged areas exhibiting signs of slight atrophy. In the 14-day postsurgery group, no white patches were observed; however, the morphology of the entire brain had been altered. The damaged areas were more severely atrophic, and the structures were more loosely connected than in the 7-day group. In the 1-day group, red-stained thalamic areas indicated that these regions had not sustained any damage; however, analysis of the remaining groups indicated that white areas had appeared by the 7th day, suggesting changes to the morphology of neurons in the thalamus. Analysis of rat brain sections using Image J software revealed that the area of infarction was most severe at the 7th day after the surgery (Figure 3(b)).

### 3.3. Functional Manifestation of Brain Nissl Bodies and Myelin

**Figure 4:**
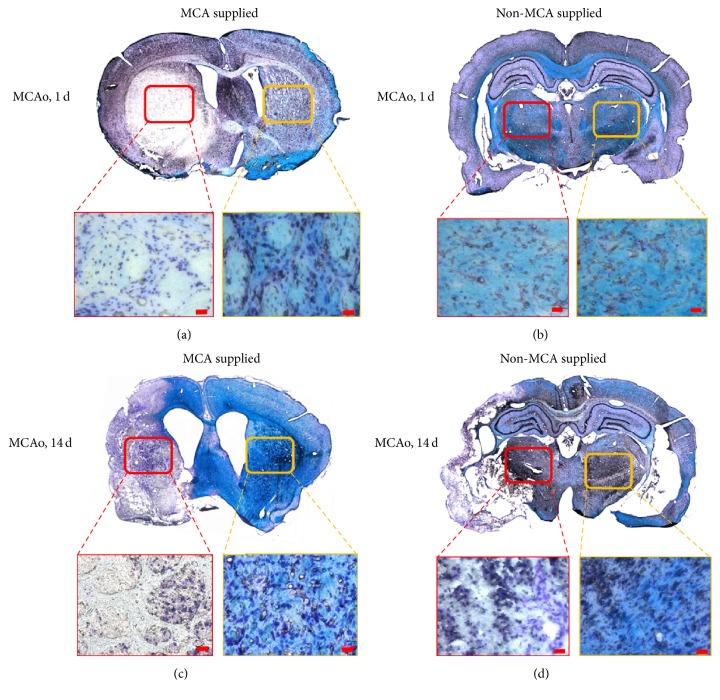
Luxol fast blue and cresyl echt violet staining at 1 and 14 days after MCAo. (a, b) Day 1 after MCAo. (c, d) Day 14 after MCAo. (a, c) Images of the brain indicating areas supplied by the MCA. The red box designates the putamen on the damaged side, and the yellow box designates the putamen on the control side. (b, d) Images of brain areas not supplied by the MCA. The red box designates the thalamic nuclei on the damaged side, and the yellow box designates the thalamic nuclei on the control side. The amount of myelin (blue dots) in the putamen on the damaged side exhibited a decrease in day 1 and day 14 groups. No difference was observed in the amount of thalamic myelin between the affected and control sides in day 1 group. In the 14-day group, the amount of myelin in the thalamus was remarkably reduced on the damaged side compared to the control side, indicating the occurrence of transneuronal degeneration. MCAo: middle cerebral artery occlusion.

Luxol fast blue staining is commonly used to identify the myelin coating of neural axons. Myelin exhibits a blue color after staining, while other regions remain transparent. Cresyl echt violet stain is used to identify nerve cells and Nissl bodies, which exhibit a magenta color. On days 1 and 14 after MCAo surgery, brain sections were stained with Luxol fast blue and cresyl echt violet (Figure 4). On day 1 after surgery, the myelin of the brain regions supplied by the MCA, including the putamen, had degenerated completely even though the nerve cells themselves remained. At this stage, no change was observed in the thalamus; both myelin and nerve cells were present. Samples taken from rats euthanized on the 14th day after surgery also exhibited an absence of myelin in the brain regions supplied by the MCA.

### 3.4. Immunohistochemistry

**Figure 5:**
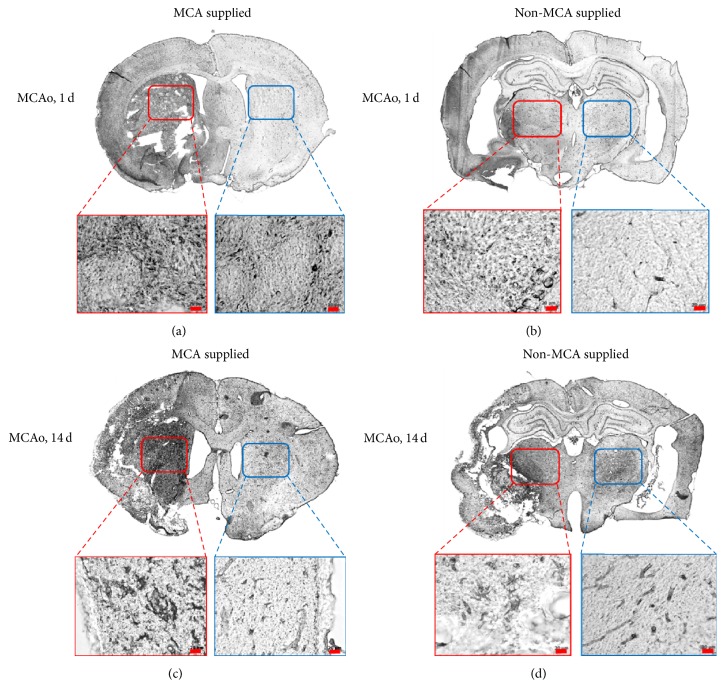
Expression of glial fibrillary acidic protein (GFAP) on day 1 and day 14 after MCAo. (a, b) Day 1. (c, d) Day 14. (a, c) Images of the brain regions supplied by the MCA. The red box designates the putamen on the damaged side. The blue box designates the putamen on the control side. (b, d) Images of brain areas not supplied by the MCA. The red box designates the thalamic nuclei on the damaged side. The blue box designates the thalamic nuclei on the control side. Damaged areas stained positive for GFAP in the 1-day group. GFAP was expressed in the putamen, but not in the thalamus, indicating that neurons in the thalamic regions were not affected at 1 day after surgery. Damaged areas remained positive for GFAP in the 14-day group, though additional staining was also observed in the thalamic region, indicating that transneuronal degeneration and subsequent increases in GFAP expression occurred in the thalamus despite not being directly supplied by the MCA. MCA: middle cerebral artery.

**Figure 6:**
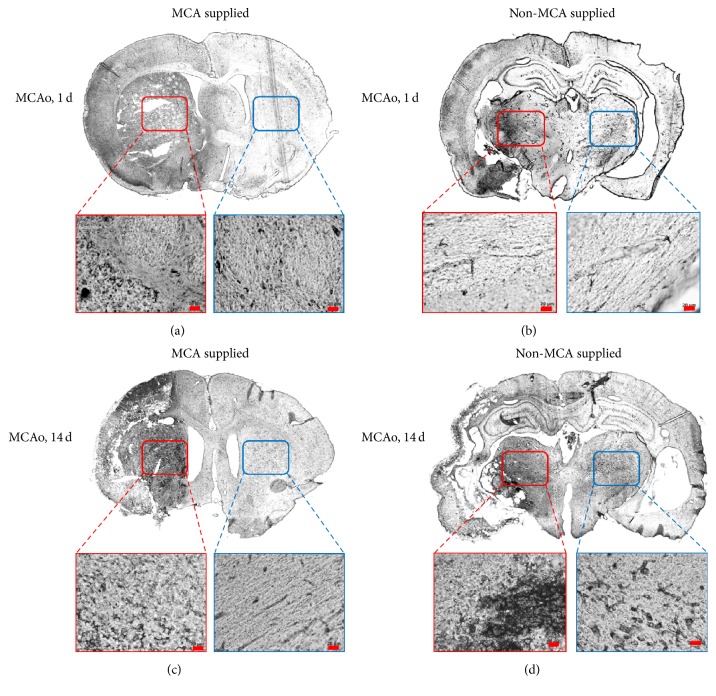
Expression of vascular endothelial growth factor (VEGF) on day 1 and day 14 after MCAo. (a, b) Day 1. (c, d) Day 14. (a, c) Images of the brain regions supplied by the MCA. The red box designates the putamen on the damaged side. The blue box designates the putamen on the control side. (b, d) Images of brain areas not supplied by the MCA. The red box designates the thalamic nuclei on the damaged side. The blue box designates the thalamic nuclei on the control side. Neurons in the damaged area stained positive for VEGF in the 1-day group, though differences in expression were observed in the thalamus and putamen. Staining results indicate that angiogenesis may have occurred in the damaged area 1 day following occlusion. In the 14-day group, neurons at the damaged site also stained positive for VEGF, though staining was also observed in the thalamus—a result remarkably different from that observed in the 1-day group. Staining results indicate that, by 14 days after surgery, levels of VEGF expression and angiogenesis may have increased in the thalamus. MCAo: middle cerebral artery occlusion.

**Figure 7:**
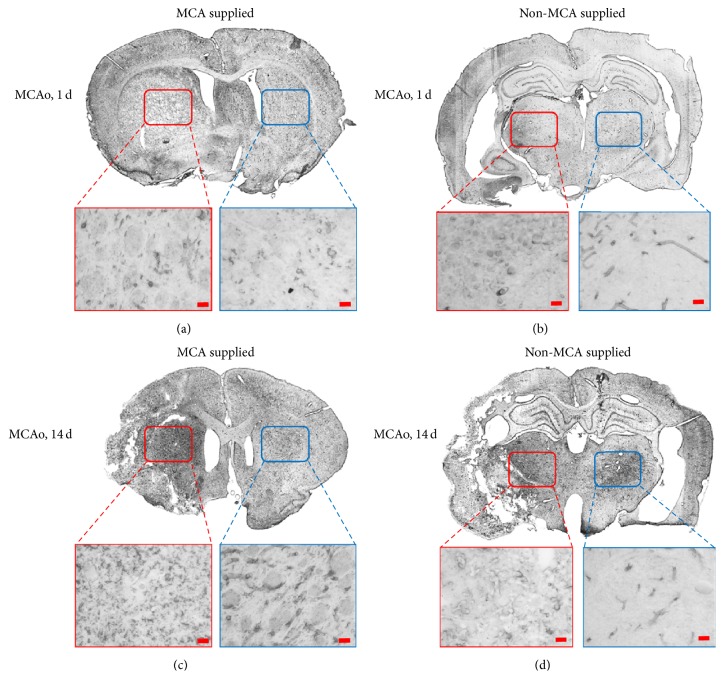
Expression of myelin basic protein (MBP) on the first day after MCAo. Staining results in (a, b) day 1 and (c, d) day 14 groups. (a, c) Images of the brain regions supplied by the MCA. The red box designates the putamen on the damaged side. The blue box designates the putamen on the control side. (b, d) Images of brain areas not supplied by the MCA. The red box designates the thalamic nuclei on the damaged side. The blue box designates the thalamic nuclei on the control side. No expression of MBP was observed at the damaged site in the 1-day group, and there were no differences in MBP expression between the putamen and thalamus. In the 14-day group, MBP expression was detected both at the damage site and in the thalamus. Again, no differences were observed between the putamen and thalamus with regard to MBP expression. The staining results indicate that levels of MBP expression increased by the 14th day following occlusion and that remyelination may have occurred at the damaged site.

Staining results at days 1 and 14 after surgery revealed that the levels of glial fibrillary acidic protein (GFAP) (Figure 5) and vascular endothelial growth factor (VEGF) (Figure 6) increased remarkably in the damaged areas. Gliosis was observed in both the 1-day and 14-day groups. Astrocyte proliferation also increased greatly in the damaged areas in these groups (Figure 5). However, certain areas, such as the thalamic nucleus in charge of movement, remained unaffected between day 1 and day 14 after surgery. Marked positive staining for myelin basic protein (MBP) (Figure 7) was observed at day 14 after surgery.

## 4. Discussion

MCAo in rats is commonly used to model focal brain ischemia in humans. Studies have indicated that stroke survivors usually exhibit chronic disability that tends to worsen with age. In the present study, no changes were observed in the anterolateral region of the thalamus following MCAo. Contralateral circling and bending were also observed in the 1-day group, indicating that MCAo did not affect the animals' ability to move. Previous research has also revealed that damage to the nigrostriatal system following MCAo causes laboratory animals to exhibit circling behavior [16]. However, the authors of this study reported changes in motor function over time. Therefore, the movement behavior test and evaluation proposed by Bederson utilized in the present study may be ill-suited to observing the long-term postoperative behavioral patterns of rats [13].

In addition, TTC staining revealed that the cerebral ischemic area was larger in the 7-day postsurgery group than in the remaining groups. Mitochondrial activity also seemed to recover by the 14th day after MCAo surgery, though the brains of rats in the 14-day group were more atrophic than those of the 1-day and 7-day groups. Though TTC staining remains the most common method used for determining the success of cerebral ischemia surgery due to its effectiveness and convenience [17], research has indicated that the results of TTC staining may be affected by the length of occlusion time [18].

Luxol fast blue and cresyl violet staining revealed that the tissue structure of the thalamus was loose in the 14-day group when compared to that of the remaining groups and that the myelin sheath in the same region was remarkably reduced when compared to that of the 1-day group. These results confirm that the thalamic region was affected indirectly by the MCAo, and the self-regeneration might occur by the 14th day after surgery.

In the central nervous system, astrocytes are relatively large glial cells with multiple functions that accumulate in areas where neurons have sustained damage. Glial cell proliferation and neuron loss in certain brain areas have been observed in a number of neurodegenerative disorders. Previous studies have reported widespread areas of necrosis 1 day following MCAo, with loose connective tissue forming a matrix border along the site of the infraction after the occlusion has been in place for 7–14 days. Moreover, the ischemic area contained a large number of astrocytes expressing GFAP [19]. The immunohistochemical staining results of the present study align with these previously published results.

Brain tissue is very sensitive to ischemia and anoxia. Following cerebral ischemia, the neuronal damage is relatively restricted in mild cases. However, when complete and persistent ischemia occurs, various neurons, glial cells, and endothelial cells inside the ischemic region undergo necrosis. After MCAo, brain tissue in the central necrotic area and surrounding ischemic penumbra become compromised. In the necrotic area, brain cells die due to complete ischemia. However, evidence from the present study seems to suggest that collateral circulation continues to supply some amount of blood to the ischemic penumbra, given the amount of viable neurons. If circulation can be restored rapidly enough to sustain brain metabolism, nerve cells may survive and recover their function. Therefore, protection of these irreversibly damaged neurons is the key to treatment of acute cerebral infarction.

The present study successfully established a rat model of cerebral ischemia and further explored long-term alterations in neural structure after cerebral infarction. VEGF was highly expressed in the damaged areas of the brain in both the 1-day and 14-day groups, indicating the occurrence of angiogenesis. Previous studies have documented the neurotrophic and neuroprotective effects of VEGF, in addition to its potential role in stimulating adult neurogenesis [20]. The expression of GFAP is also related to the rehabilitation process following brain injury. Therefore, the interaction between GFAP and VEGF may provide an avenue for future strategies for the treatment of chronic cerebral infarction.

Furthermore, the relative levels of MBP provide more insight into changes that occur following MCAo, as increased expression of MBP indicates remyelination while reduced expression indicates demyelination. In the present study, marked increases in MBP expression were observed in the 14-day group when compared to levels of the 1-day group, indicating the potential involvement of MBP in the proliferation of oligodendrocyte progenitor cells [21]. The increased expression of GFAP, VEGF, and MBP may help the brain to recover temporarily and offset its loss of function and thus may be the key to future treatment for patients who have experienced a stroke.

## 5. Conclusions

The results of the present study provide evidence of myelin loss in the thalamus, changes in VEGF, and glial responses following middle cerebral artery occlusion in a rate model of ischemia, indicative of neuronal degeneration. In addition, the increased expression of GFAP and VEGF is observed in the damaged and surrounding areas and may help the brain to recover temporarily and offset losses of function. The rat model for cerebral ischemic stroke established in the present study may be utilized in future research regarding the prevention of transneuronal degeneration before irreversible damage occurs and aid in the development of new treatment strategies for patients with stroke.

## Figures and Tables

**Table 1:** The scoring criteria for locomotor activity test in rats. Rats were subjected to tail suspension and open field tests following middle cerebral artery occlusion and scored as indicated here.

Score	Behavior
0	Rats with normal behavior
1	The forelimb and body of the rat bend consistently to the side contralateral to that of the damaged brain hemisphere
2	The rat can move to both sides, but when its tail is held, it can only circle to the side contralateral to that of the damaged brain hemisphere
3	The rat can only go move in circles in the direction of the side contralateral to that of the damaged brain hemisphere
4	The rat has difficulty in walking or loses its ability to walk
